# Supplementary material for: The genome sequence of the Star Sedge, Carex echinata Murray (Poales: Cyperaceae)
Source: Wellcome Open Res. 2026 Oct 13;11:866. [Version 1] doi: 10.12688/wellcomeopenres.28117.1 (PMC13695709; doi:10.12688/wellcomeopenres.28117.1)
Supplement: Peer review history

# Reviewer report: Reviewer response for version 1

**DOI:** 10.21956/wellcomeopenres.30936.r174256

**Published:** 2026-10-24

**Authors:** MD MAFIZUR Rahman

**Affiliations:** 1 Islamic University Kushtia, Kushtia, Bangladesh

**Title:** **The genome sequence of the Star Sedge, Carex echinata Murray (Poales: Cyperaceae)**

**Genome Note in Wellcome Open Research**

 This manuscript presents a high-quality, chromosome-level reference genome assembly for the Star Sedge ( *Carex echinata*), generated as part of the Darwin Tree of Life (DToL) Project. Nevertheless, the manuscript is well-written and technically sound; the following minor points could be addressed to enhance clarity:

 Minor concerns:

1. The authors should focus on the significance of Star Sedge ( *Carex echinata*) genome in Introduction section, specifically focus on plant evolutionary biology, comparative genomics within Cyperaceae, and regional biodiversity conservation.
2. The authors should clarify that Figure 5’s snail plot caption references the older poales_odb10 dataset (80.04% complete), whereas the main text and Table 4 correctly highlight the updated poales_odb12 dataset (97.9% complete). Updating the figure label or clarifying the lineage version difference in the text/caption footnote would prevent minor reader confusion.  
3. Though the authors mention in background references a sporophytic count of 2n = 56 or 58. But a brief sentence in the genome report noting that the 2n = 29 chromosome-level pseudomolecules perfectly match the expected 2n = 58 karyotype would neatly connect the genomic output with classical cytogenetics.

Are the datasets clearly presented in a usable and accessible format, and the assembly and annotation available in an appropriate subject-specific repository?

Yes

Are sufficient details of the sequencing and extraction, software used, and materials provided to allow replication by others?

Yes

Are the rationale for sequencing the genome and the species significance clearly described?

Partly

Are the protocols appropriate and is the work technically sound?

Yes

Reviewer Expertise:

Molecular Biology, Genomics, Comparative Genomics, Pest and Pathogens genomics

I confirm that I have read this submission and believe that I have an appropriate level of expertise to confirm that it is of an acceptable scientific standard.

# Reviewer report: Reviewer response for version 1

**Published:** 2026-10-13

**Authors:** Automated Benchmarking

**Affiliations:** 1 Wellcome Sanger Institute, Hinxton, UK

As this article is a Genome Note, Automated Benchmarking has been used to determine whether the reported assembly passes four defined checks established by the Earth BioGenome Project: the raw and assembled data are publicly available, the Earth BioGenome Project three-part metric is met, the k-mer completeness exceeds 90%, and the BUSCO score passes the stated threshold. For Carex echinata (lpCarEchi2), all four checks passed. Data available: pass. ENA study PRJEB88758, raw-read accessions ERR14886543 and ERR14895954, and assembly accessions GCA_965279795.1 and GCA_965279845.1 are public. Earth BioGenome Project three-part metric: pass. Haplotype 1 is reported as 7.C.Q66, meeting the 6.C.Q40 reference standard. k-mer completeness: pass. The combined haplotype assemblies have 99.27% k-mer completeness, exceeding the Earth BioGenome Project target of 90%. BUSCO: pass. Haplotype 1, which is proposed as the reference assembly for this species, has 97.9% complete BUSCOs (94.7% single-copy and 3.2% duplicated). BUSCO software version: 6.1.0; lineage dataset: poales_odb12. It meets the plant-specific target of greater than 90% complete BUSCOs. This decision uses values reported in the Genome Note and public accession records.

Reviewer Expertise:

NA

I confirm that I have read this submission and believe that I have an appropriate level of expertise to confirm that it is of an acceptable scientific standard.
